# Evaluation of active inflammation, chronic structural damage, and response to treatment of sacroiliitis in axial spondyloarthritis using the Spondyloarthritis research consortium of Canada scoring system

**DOI:** 10.1186/s12891-022-05609-x

**Published:** 2022-07-08

**Authors:** Yimeng Zhang, Zikang Guo, Ying Zhan, Jin Qu, Xinwei Lei

**Affiliations:** 1 Department of Radiology, First Central Clinical College, Tianjin Medical University, Tianjin, 300070 China; 2 Department of Radiology, Tianjin First Central Hospital, Tianjin, 300192 China

**Keywords:** Magnetic resonance imaging, Axial spondyloarthritis, Spondyloarthritis research consortium of Canada, Sacroiliac joint, Treatment response

## Abstract

**Background:**

Axial spondyloarthritis (axSpA) is a chronic inflammatory rheumatic disease affecting the spine and sacroiliac joints. To investigate whether there are differences in inflammatory and chronic structural damages, as assessed by a semiquantitative MRI scoring method, between non-radiographic axial spondyloarthritis (nr-axSpA) and ankylosing spondylitis (AS) patients with active inflammation at baseline, and to evaluate the treatment response in these patients after 3 months of tumor necrosis factor-alpha (TNF-α) inhibitor treatment.

**Methods:**

Fifty-eight axSpA patients with active inflammation were included in the study. The patients were divided into nr-axSpA group and AS group. MRI examinations of the sacroiliac joints were performed before and after treatment. Inflammatory and structural damages in these patients were assessed using the established Spondyloarthritis Research Consortium of Canada (SPARCC) inflammation and sacroiliac joint structural (SSS) scoring methods, which are two MRI-based scoring methods. The SPARCC score, SSS score, erythrocyte sedimentation rate (ESR), and C-reactive protein (CRP) level were compared between the two groups.

**Results:**

At baseline, SPARCC scores for patients in the nr-axSpA and AS groups did not differ significantly (*P* > 0.05); however, SSS scores for fat metaplasia, erosion, and backfill for patients in the AS group were significantly higher (*P* < 0.001). Compared with baseline, SPARCC scores were significantly decreased in both groups after treatment (*P* < 0.001); however, after treatment, no statistically significant difference was found regarding SPARCC scores between the AS and nr-axSpA groups. Compared with baseline, a significant increase in the SSS scores for fat metaplasia and backfill (*P* < 0.001) and a significant decrease in the SSS scores for erosion (*P* < 0.001) were observed in all axSpA patients. Changes in the SPARCC score was inversely correlated with the changes in the SSS score for fat metaplasia (*r* = − 0.634, *P* < 0.001). Changes in the SSS score for backfill were positively correlated with the changes in the SSS score for fat metaplasia (*r* = 0.277, *P* < 0.05) and inversely correlated with those for erosion (*r* = − 0.443, *P* < 0.001).

**Conclusion:**

The SPARCC and SSS scoring systems can be used to assess inflammatory and chronic structural damages as well as treatment responses in patients with axSpA. More severe structural damages were seen in AS patients. TNF-α inhibitor treatment for 3 months could effectively reduce inflammation in axSpA patients.

**Supplementary Information:**

The online version contains supplementary material available at 10.1186/s12891-022-05609-x.

## Introduction

Axial spondyloarthritis (axSpA) is a chronic inflammatory rheumatic disease affecting the spine and sacroiliac joints. It can be categorized as non-radiographic axial spondyloarthritis (nr-axSpA) and radiographic axial spondyloarthritis (r-axSpA); the latter is also termed ankylosing spondylitis (AS) [1]. Although patients with nr-axSpA and AS share many clinical symptoms and experience a similar burden of disease [2–4], they also differ in several ways, including disease duration, gender predilection, and levels of inflammatory markers [5, 6]. However, their differences on magnetic resonance imaging (MRI) remain unclear.

MRI, a noninvasive and sensitive technique, is an established method for diagnosing and managing axSpA [7]. Inflammation is an indicator for axSpA progression at the early stage, which is common in the sacroiliac joints, resulting in sacroiliitis. Subchondral bone marrow edema (BME) on MRI is thought to reflect active sacroiliitis and is thus used as an early diagnostic marker and part of the classification criteria for axSpA [7, 8]. Recently, several studies have proposed that chronic structural damage might provide additional information for assessment of axSpA. Chronic structural damages in the sacroiliac joints of axSpA patients include subchondral sclerosis, fat metaplasia, erosion with or without backfill, and resultant ankylosis. Fat metaplasia and backfill are early post-inflammatory changes that most likely reflect the early stage of bone remodeling [9–11], for which the histopathology is unknown. Specifically, backfill is one of the characteristics of new bone formation on MRI of axSpA [12–15].

Systematic MRI-based scoring systems for inflammatory and structural damages in the sacroiliac joints have been developed and validated [15]. The Spondyloarthritis Research Consortium of Canada (SPARCC) scoring method is the most popular imaging standard for inflammation, with high accuracy and reproducibility [16–18]. Previous studies have shown a correlation between SPARCC scores and clinical disease activity. The SPARCC sacroiliac joint structural (SSS) scoring method is a new method that assesses a broad spectrum of structural damages [19, 20]. However, assessment of this new scoring system in clinical trials has been limited.

This study assessed the differences in the inflammatory and structural damages displayed on MRI between nr-axSpA and AS patients with active inflammation at baseline; furthermore, it investigated the response of axSpA patients to tumor necrosis factor-alpha (TNF-α) inhibitor treatment using a semiquantitative MRI-based scoring system.

## Materials and methods

### Methods

This study was a longitudinal cohort study consisting of patients aged 18–45 years who had symptoms for more than 3 months but less than 5 years. Patients were included if they were diagnosed with axSpA and had active inflammation on MRI. These patients were diagnosed according to the 2009 Assessment of SpondyloArthritis International Society (ASAS) criteria (either nr-axSpA or AS if patients had sacroiliitis on X-ray meeting the Modified New York Criteria) [7, 21].

In total, 58 patients with axSpA who had baseline and 3-month after-treatment (i.e., TNF-α inhibitor) scans were evaluated systematically according to a standardized protocol. Because of the study’s retrospective nature, flexible windows of ±1 month for MRI scans were permitted. These patients were assessed to be suitable for TNF-α inhibitor treatment by physician and had not previously been treated with other biological agent. For TNF-α inhibitor treatments, patients received either etanercept or adalimumab. This study was approved by the Institutional Review Board of Tianjin First Central Hospital Medical Ethics Committee (2021N143KY), and the requirement of informed consent was waived because the study was retrospective. All experiments were conducted in accordance with the Declaration of Helsinki (1964), and all methods were performed following the relevant guidelines and regulations.

### MRI protocol

All patients were scanned using a 3.0 Tesla (T) MR scanner (Ingenia, Philips Healthcare, the Netherlands). All sequences were obtained by scanning the oblique coronal plane parallel to the sacrum plane. The scanning sequences and parameters were as follows: modified Dixon turbo spin echo (mDixon TSE) T1-weighted images (T1WIs) (time of repetition (TR), 462 ms; echo time (TE), 16 ms; field of view (FOV), 220 × 220 mm; slice thickness, 3 mm; slice gap, 0 mm; matrix, 276 × 210), mDixon-TSE T2-weighted images (T2WIs) (TR, 2300 ms; TE, 90 ms; FOV, 220 × 220 mm; slice thickness, 3 mm; slice gap, 0 mm; matrix, 276 × 219). After scanning, T1WIs and T2WIs were automatically reconstructed and generated.

### Definition of BME and structural damage

BME appears as the low-intensity signal on T1WIs and high-intensity signal on T2WIs and is clearly present in the subchondral bone marrow [22].

Structural damages include bone erosion, fat metaplasia, backfill, and bone bridges/ankylosis [22]. All definitions are based on T1W scans. The standard definitions are as follows:

- Erosion: full-thickness loss of the dark appearance of cortical bone and loss of the normal appearance of the adjacent bone marrow.
- Fat metaplasia: the signal is brighter than normal bone marrow, which meets the following conditions: homogeneous; located in the subchondral bone; sharply defined borders.
- Backfill: increased signal at a complete loss of the cortical bone, clearly demarcated from the adjacent normal marrow by an irregular dark signal.
- Ankylosis: bone marrow signal extending between the sacral and iliac bone marrow with full-thickness loss of the dark appearance of the iliac and sacral cortical bone.

### MRI scoring methodology

All MRIs of the sacroiliac joints were evaluated for BME according to the SPARCC method [16]. First, each joint was divided into four quadrants: the superior ilium, inferior ilium, superior sacrum, and inferior sacrum. Dichotomous scoring was performed according to the presence or absence of bone marrow edema signals. Second, the depth and intensity of the lesion were assessed (a signal of the presacral vessels was defined as an intense lesion, and a signal extending at least 1 cm from the articular surface was defined as a deep lesion). The maximum score for a single coronal slice was 12, and the scoring system was repeated in six consecutive coronal slices, leading to a total score of 72. A score ≥ 2 was considered an indicator of inflammation based on MRI images [19, 20].

All MRI images of the sacroiliac joints were evaluated for fat metaplasia, erosion, backfill, and ankylosis according to the SPARCC SSS method, which was used to assess the structural lesions on T1WIs [19]. The presence or absence of lesions was scored in quadrants (for fat metaplasia and erosion) or halves (for backfill and ankylosis). Scoring was mutually exclusive for individual quadrants on individual images when assessing erosion and backfill. The maximum score for a single coronal slice was 24, and the scoring system was repeated in five consecutive coronal slices, leading to a total score of 120. The scoring ranges were as follows: fat metaplasia, 0–40; erosion, 0–40; backfill, 0–20; ankylosis, 0–20.

### Reading exercises

Readers were blinded to patient demographics and treatments. A calibration exercise was conducted in 10 cases randomly selected from the cohort using baseline and 3-month MR scans after TNF-α inhibitor treatment scored by two radiologists blinded to the time points.

### Interobserver agreement

Baseline and 3-month MR scans of the sacroiliac joint were scored independently by two radiologists, who were blinded to all clinical data.

### Statistical analysis

All data were statistically analyzed using the SPSS 26.0 software. Continuous data are expressed as means ± standard deviation (SD), and categorical data are expressed as percentages. Analyses were performed using the mean scores from the two readers. Normally distributed variables were compared using an independent t-test, and non-normally distributed variables were compared using the Mann-Whitney U test. Categorical variables were compared between groups using the chi-squared test. Associations between changes in parameters of inflammation (CRP and SPARCC score) and SSS subgroup scores were examined using the *Spearman* correlation analysis. Interobserver reliability for baseline and 3-month scores was assessed using the intraclass correlation coefficient (ICC). The correlation strengths were defined as follows: 0–0.2, poor; 0.3–0.4, fair; 0.5–0.6, moderate; 0.7–0.8, strong; > 0.8, excellent agreement. *P* < 0.05 was considered statistically significant.

## Results

### Patient characteristics

**Fig. 1:**
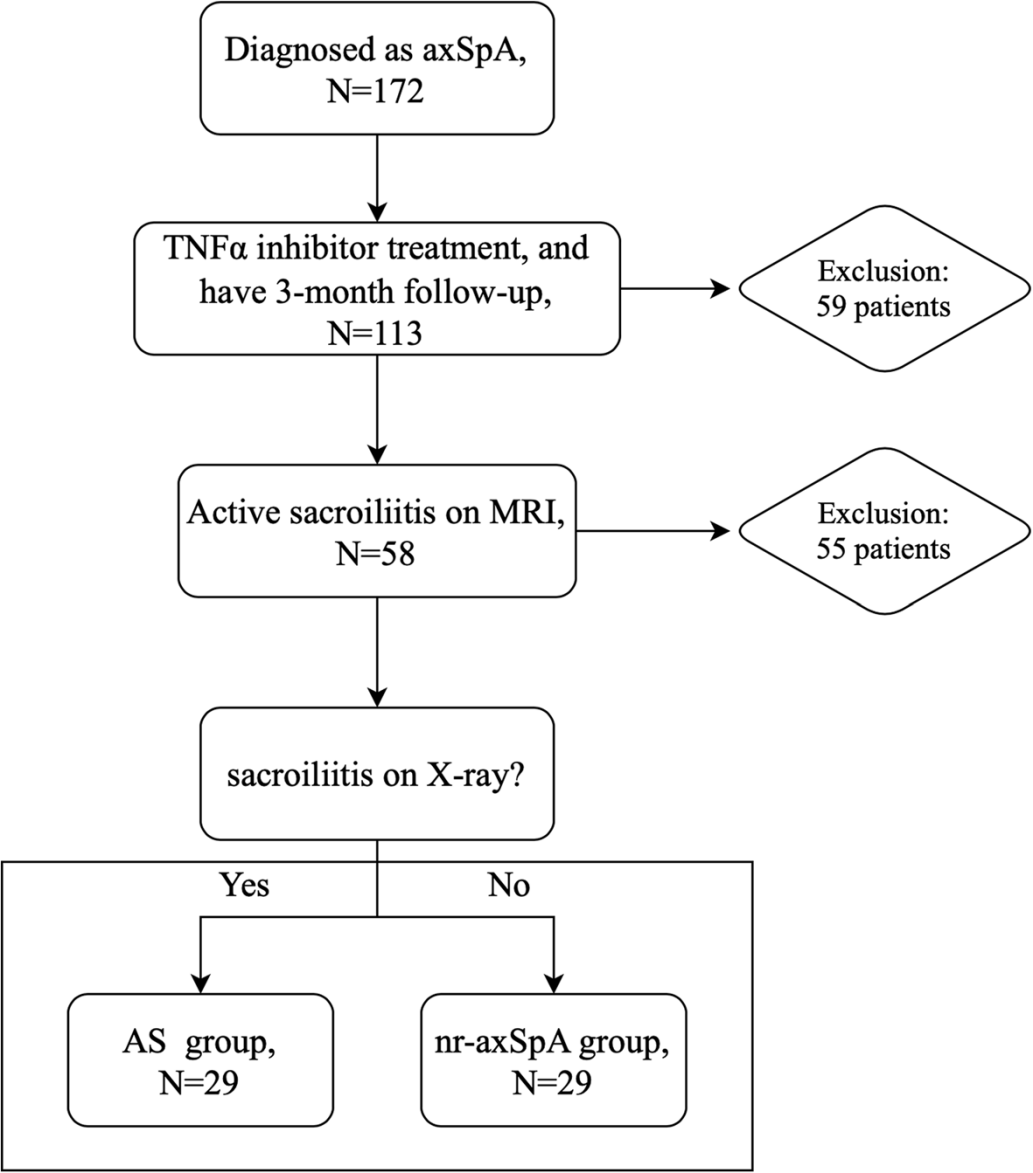
Study flow diagram. axSpA: axial spondyloarthritis; TNF-α: tumor necrosis factor-alpha; MRI: magnetic resonance imaging; AS: ankylosing spondylitis; nr-axSpA: non-radiographic axial spondyloarthritis

A total of 58 patients were evaluated. The study flow diagram is presented in Fig. 1. The mean ± SD age of the patients was 25.69 ± 6.79 years. The mean ± SD CRP concentration was 17.52 ± 29.41 mg/L, and the mean ± SD ESR concentration was 24.43 ± 23.83 mm/h. The characteristics of the patients are presented in Table 1.

**Table 1:** Patient characteristics and disease parameters

	Total axSpA	nr-axSpA(*n* = 29)	AS(*n* = 29)	*P*-value
Age (years)	25.69 ± 6.79	25.00 ± 6.00	27.00 ± 7.40	0.198
Male(n,%)	41(70.69)	18(62.09)	23(79.31)	0.149
HLA-B27(n,%)	33(56.90)	13(44.83)	20(68.97)	0.248
Baseline				
ESR (mm/h)	24.43 ± 23.83	18.60 ± 17.80	30.31 ± 27.70	0.065
CRP (mg/L)	17.52 ± 29.41	9.33 ± 22.41	25.40 ± 33.41	0.053
SPARCC	24.79 ± 17.13	22.90 ± 15.23	26.69 ± 18.91	0.602
SSS	26.93 ± 20.26	14.26 ± 11.70	39.60 ± 19.13	< 0.001

*ESR* Erythrocyte sedimentation rate, *CRP* C-reactive protein, *SPARCC* Spondyloarthritis Research Consortium of Canada, *SSS* SPARCC Sacroiliac Joint Structural Scores

### Inflammatory and structural damage at baseline

At baseline, 38 patients (65.52%) had BME on both sides; 33 patients (56.90%) had BME with a depth greater than 1 cm. Almost all patients (93.1%) had BME on the sacral side of the sacroiliac joints, either unilateral or bilateral. Fat metaplasia was found in 51 patients (87.93%), of which 41 (80.39%) were bilaterally affected. Erosion was found in 53 patients (91.38%), and backfill was found in only 22 patients (37.93%).

The mean ± SD SPARCC score was 24.79 ± 17.13. Most patients had a score of > 0 for fat metaplasia, erosion, and backfill in the sacroiliac joints. No patients showed ankylosis at baseline. The mean ± SD SSS was 26.93 ± 20.26. A higher SPARCC score was observed in the AS group than in the nr-axSpA group; however, this difference was not significant. Significant differences were found in the SSS for fat metaplasia, erosion, and backfill at baseline (Tables 1 and 2).

**Table 2:** SPARCC and SSS values at baseline and 3-month follow-up in 58 patients

	Total axSpA	nr-axSpA(*n* = 29)	AS(*n* = 29)	*P*-value
SPARCC				
Baseline	24.79 ± 17.13	22.90 ± 15.23	26.69 ± 18.91	0.602
3-month	7.74 ± 9.18**	6.91 ± 7.04**	8.57 ± 10.99**	0.755
Fat metaplasia				
Baseline	14.89 ± 12.14	8.36 ± 9.82	21.41 ± 10.75	< 0.001
3-month	21.09 ± 11.81**	14.02 ± 9.20*	28.17 ± 9.78*	< 0.001
Erosion				
Baseline	9.58 ± 8.53	5.69 ± 4.73	13.47 ± 9.72	0.001
3-month	4.65 ± 4.88**	3.19 ± 3.05*	6.10 ± 5.90**	0.061
Backfill				
Baseline	2.47 ± 4.13	0.21 ± 0.63	4.72 ± 4.87	< 0.001
3-month	3.78 ± 5.11**	1.10 ± 2.05*	6.47 ± 5.83	< 0.001

Comparison of baseline and 3-month follow-up, **: *P* < 0.001 *: *P* < 0.05

**Fig. 2:**
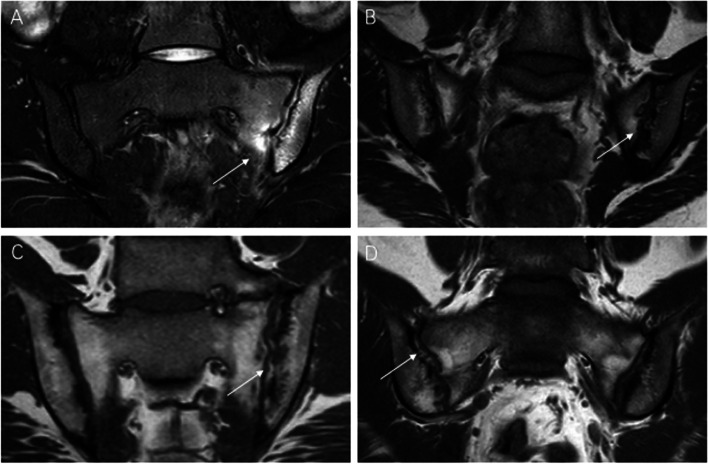
MRI findings of active inflammation and chronic structural damage in axSpA. **A** Bone marrow edema on the left side of sacroiliac joints. **B** Erosion of the left iliac bone and sacral bone. **C** Fat metaplasia in the bilateral sacroiliac joints. **D** Backfill in the right sacroiliac joints. MRI: magnetic resonance imaging; axSpA: axial spondyloarthritis

Examples of inflammatory and chronic structural damages identified based on MRI images are shown in Fig. 2.

### Changes in inflammatory and structural damage after 3 months of treatment

**Fig. 3:**
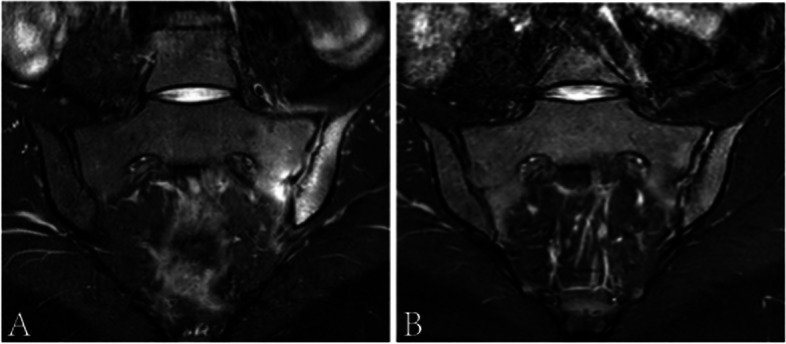
Inflammation changes at baseline (**A**: SPARCC score 34) and 3-month follow-up (**B**: SPARCC score 14)

**Fig. 4:**
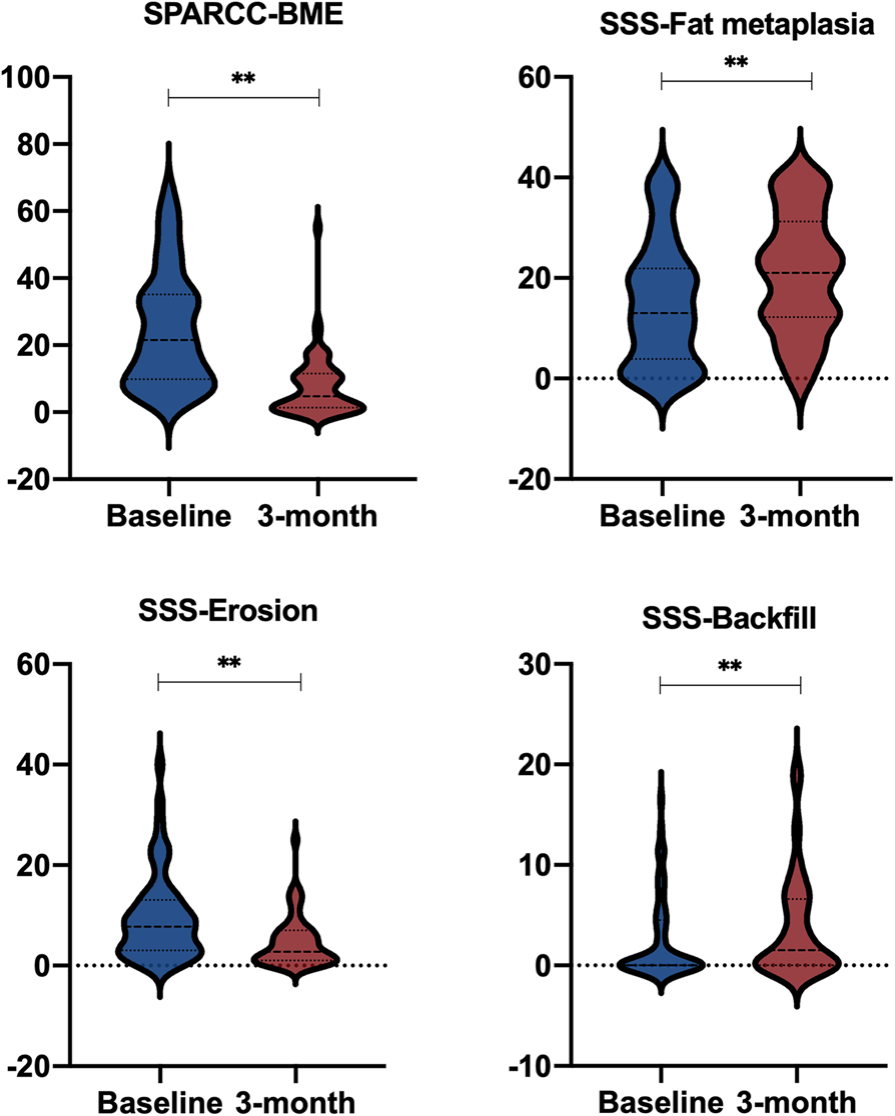
Changes (increase or decrease) in SPARCC score for BME and SSS for fat metaplasia, erosion, and backfill in patients with axSpA (including nr-axSpA and AS)

**Fig. 5:**
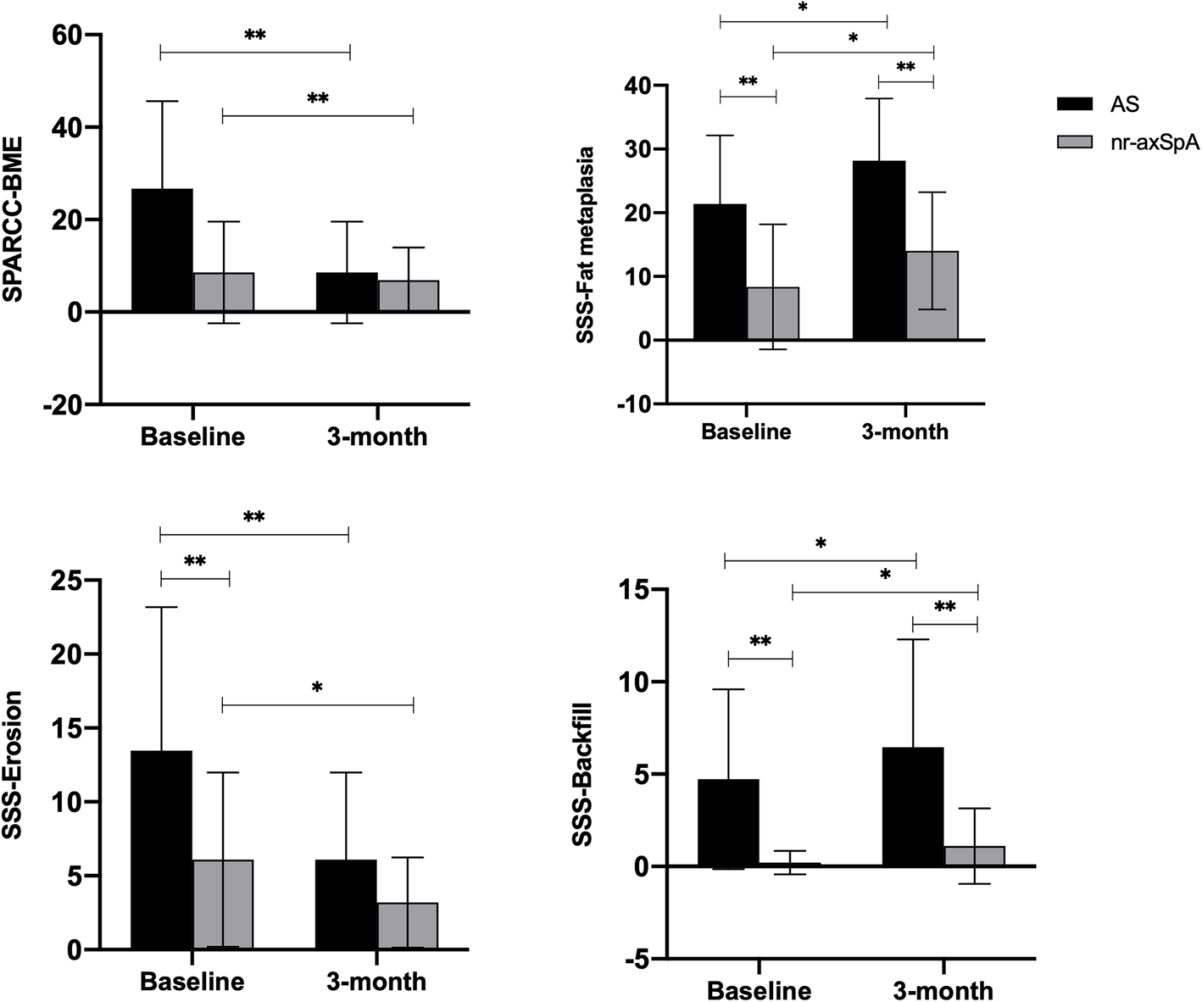
Differences in the SPARCC score for BME and the SSS for fat metaplasia, erosion, and backfill between AS and nr-axSpA groups at baseline and 3-month follow-up

Compared with baseline, SPARCC scores were significantly decreased after treatment in the AS group (*P* < 0.001) (Table 2). Similar outcomes were observed in the nr-axSpA group (*P* < 0.001) (Table 2 and Fig. 3). However, no statistically significant difference was found regarding SPARCC scores between the AS and nr-axSpA groups after treatment. Regarding chronic structural damage, a significant increase in the SSS score for fat metaplasia and backfill (both *P* < 0.001) and a decrease in SSS for erosion (*P* < 0.001) were observed in axSpA patients after treatment. The variations in the SPARCC scores for BME and the SSS scores for fat metaplasia, erosion, and backfill in axSpA patients, as well as the difference between the nr-axSpA and AS groups, are shown in Figs. 4 and 5.

### Correlations between changes in inflammatory and structural damage

The changes in SPARCC scores were inversely correlated with the changes in the SSS scores for fat metaplasia (*r* = − 0.634, *P* < 0.001) and backfill (*r* = − 0.307, *P <* 0.05). The changes in the SSS score for backfill were positively correlated with the changes in fat metaplasia (*r* = 0.277, *P <* 0.05) and inversely with the changes in erosion (*r* = − 0.443, *P* < 0.001) (Table 3).

**Table 3:** Associations between changes in inflammatory and structural damage

	SPARCC- BME	SSS-Fat metaplasia	SSS-Erosion	SSS-Backfill
CRP				
*r*	−0.068	0.074	0.009	0.050
*P*	0.612	0.582	0.949	0.708
SPARCC-BME				
*r*	–	−0.634	0.080	− 0.307
*P*		< 0.001	0.550	0.019
SSS-Fat metaplasia				
*r*	–	–	−0.129	0.277
*P*			0.335	0.035
SSS-Erosion				
*r*	–	–	–	−0.443
*P*				< 0.001

Correlations were determined using Spearman correlation analysis. *CRP* C-reactive protein, *SPARCC* Spondyloarthritis Research Consortium of Canada, *BME* Bone marrow edema, *SSS* SPARCC Sacroiliac Joint Structural Scores

### Agreement between readers

For MRI evaluation, strong to excellent agreement was observed between the two readers. Excellent interobserver reliability was achieved for the identification of inflammation, erosion, and backfill at baseline and 3-month follow-up and for the identification of fat metaplasia at baseline (ICC 0.88–0.96, 0.86–0.95, 0.85–0.94, and 0.85–0.94, respectively). A strong to excellent interobserver reliability was achieved for the identification of fat metaplasia at the 3-month follow-up (ICC 0.75–0.91).

## Discussion

This semiquantitative analysis analyzed the inflammatory and structural damages based on MRI images of axSpA patients at baseline and after 3 months of treatment, which demonstrated that MRI SPARCC scoring system could be used to evaluate the inflammation and therapeutic efficacy in patients with axSpA. The results indicated that patients with AS had more severe structural damage, and TNF-α inhibitor treatment could effectively reduce inflammation in axSpA patients after 3 months.

At baseline, almost all patients had either unilateral or bilateral BME on the sacral side of the sacroiliac joints. More than half of the patients had BME that extended more than 1 cm from the sacroiliac joint space, a condition known as deep inflammatory lesions. Different levels of BME showed different courses of development during the follow-up period. This demonstrates the need for a more accurate method to assess BME, and the SPARCC scoring system, which can be used to assess disease activity, has been proposed as a suitable method [23–25]. In this study, the SPARCC scoring system provided a reference for evaluating treatment efficacy. Patients with sacroiliitis showed significantly decreased SPARCC scores after 3 months of TNF-α inhibitor treatment, indicating TNF-α inhibitor therapy is effective in treating axSpA patients. These results suggest that MRI could effectively guide clinical medication prescription. Because of its design, our study was limited to changes observed on MRI and did not compare the effects of different TNF-α inhibitors; however, this comparison will be performed in our future research. In addition, previous studies have found that structural damage in the sacroiliac joints is associated with a more severe disease phenotype with spinal progression [26, 27]. Consequently, the presence of structural damage may be used to select patients for early intervention.

MRI data from prospective observation suggest that the development of new tissue follows the resolution of inflammation in the erosion cavity. This phenomenon, termed “backfill” [9, 28], has high specificity (96%) for diagnosing axSpA [12]. We observed significant changes in fat metaplasia and backfill after 3 months of treatment. However, because the patients we selected all had active inflammation at baseline, the selectivity was high. During the same period, the evolution of erosion to backfill was evident in half of the patients after 3 months. Correlation analysis demonstrated a significant association between inflammation and fat metaplasia. Our results are consistent with those of a previous study in which treatment with etanercept was associated with a more significant reduction in erosion and an increase in backfill at 12 weeks compared with placebo [29].

Several previous studies have found that fat metaplasia and backfill are key intermediary steps in the development of new bone formation [9, 14], but the histology remains unclear. One study showed that the fat signal detected by spinal MRI of patients with AS was related to a high content of adipocytes. Therefore, the disturbance of the homeostasis between osteoblasts and osteoclasts may be a crucial reason for new bone formation [30]. In our study, no case exhibited new bone formation or ankylosis at the 3-month follow-up; possible explanations for this include the early disease stage of the selected patients, the slow development of new bone formation, and the treatment effectiveness, which slowed down the process of new bone formation. Therefore, future studies should include a longer follow-up and a control group for comparison.

This study has several limitations. First, the sample size is small; however, despite the relatively limited sample, this work offers valuable insights. Second, because of its retrospective nature, the timing of MRI examinations was not similar in all patients. Third, the study could not analyze the prognostic implications of new bone formation. Considering these limitations, larger studies performed over a longer follow-up period should be conducted.

## Conclusion

In conclusion, MRI-based scoring methods such as the SPARCC and SSS can be used to assess inflammatory and chronic structural damages in axSpA as well as patients’ responses to treatment. More severe structural damage was observed in AS patients with active inflammation at baseline. TNF-α inhibitor treatment effectively reduced inflammation in axSpA patients after 3 months.

## Supplementary Information

**Additional file 1.**

**Additional file 2.**

**Additional file 3.**

## Data Availability

The datasets supporting the conclusions of this article are included within the additional files.

## Abbreviations

AxSpA: Axial spondyloarthritis
Nr-axSpA: Non-radiographic axial spondyloarthritis
AS: Ankylosing spondylitis
MRI: Magnetic resonance imaging
TNFα: Tumor necrosis factor-alpha
SPARCC: Spondyloarthritis Research Consortium of Canada
SPARCC SSS: SPARCC Sacroiliac Joint Structural Score
BME: Bone marrow edema
ASAS: Assessment of SpondyloArthritis international Society
T1WI: T1-weighted images
T2WI: T2-weighted images
T: Tesla
FOV: Field of view
TE: Echo time
TR: Time of repetition
SD: Standard deviations
CRP: C-reactive protein
ESR: Erythrocyte sedimentation rate
ICC: Intraclass correlation coefficient
